# The Relationship between Symptom Severity and Low Vitamin D Levels in Patients with Schizophrenia

**DOI:** 10.1371/journal.pone.0165284

**Published:** 2016-10-27

**Authors:** Süheyla Doğan Bulut, Serdar Bulut, Dicle Görkem Atalan, Tonguç Berkol, Eda Gürçay, Türker Türker, Çiğdem Aydemir

**Affiliations:** 1 Psychiatry Department, Dışkapı Yıldırım Beyazıt Teaching and Research Hospital, Ankara, Turkey; 2 Psychiatry Department, Yenimahalle Teaching and Research Hospital, Yenimahalle, Ankara,Turkey; 3 Psychiatry Department, Erenköy Teaching and Research Hospital for Neurological and Psychiatric Disorders, İstanbul, Turkey; 4 Psychiatry Department, Bakırköy Mazhar Osman Teaching and Research Hospital for Neurological and Psychiatric Disorders, İstanbul, Turkey; 5 Physical Medicine and Rehabilitation Department, Dışkapı Yıldırım Beyazıt Teaching and Research Hospital, Ankara,Turkey; 6 Public Health Department, Gülhane Military Medical School, Ankara, Turkey; 7 Psychiatry Department, Numune Teaching and Research Hospital, Ankara, Turkey; Chiba Daigaku, JAPAN

## Abstract

**Background:**

In recent years, the relationship between schizophrenia and environmental factors has come into prominence. This study investigated the relationship between vitamin D levels and the positive and negative symptoms of schizophrenia by comparing vitamin D levels between patients with schizophrenia and a healthy control group.

**Methods:**

The study included 80 patients diagnosed with schizophrenia and 74 age- and sex-matched controls. The Scale for the Assessment of Negative Symptoms (SANS) and the Scale for the Assessment of Positive Symptoms (SAPS) were used to evaluate symptom severity. The 25-hydroxyvitamin D (25OHD) levels of all subjects both patients and healthy controls were analyzed in relation to measurements of symptom severity.

**Results:**

There were no significant differences between the groups in terms of age, sex, or physical activity. Their mean 25OHD levels were also similar (23.46±13.98ng/mL for the patient group and 23.69±9.61ng/mL for the control group). But when patients with schizophrenia were grouped based on their vitamin D levels, the results indicated a statistically significant differences between their vitamin D levels and their total SANS, affective flattening, and total SAPS, bizarre behavior and positive formal thought disorder scores (p = 0.019, p = 0.004, p = 0.015, p = 0.009 and p = 0.019, respectively). There is a negative correlation between 25OHD levels and SANS total points (r = -0.232, p = 0.038); a negative correlation for attention points (r = -0.227, p = 0.044) and negative correlation with positive formal thoughts (r = -0.257, p = 0.021).

**Conclusion:**

The results of this study show a relationship between lower levels of vitamin D and the occurrence of positive and negative symptoms, along with increased severity of symptoms at lower levels of vitamin D, suggesting that treatment for schizophrenia should include assessment of patients’ vitamin D levels. We recommend that patients with schizophrenia should be assessed with regard to their vitamin D levels.

## Introduction

In recent years, studies of neuroendocrine issues related to schizophrenia have increased rapidly. Findings that environmental factors like vitamin D deficiencies in individuals born during the winter, living in specific geographical places, or resulting from prenatal infections, have resulted in a focus on the relationship between schizophrenia and vitamin D [1].

Vitamin D is a steroidal hormone that can be synthesized in the skin with ultraviolet B and obtained from eating certain foods [2]. Vitamin D2 (ergocalciferol) and vitamin D3 (cholecalciferol) are the two important forms of vitamin D and both can be obtained through one’s diet. In fact, only Vitamin D3 can be synthesized in the skin with ultraviolet B [3]. Both forms of vitamin D are bound with proteins and transformed in the liver into 25OHD. 25OHD is the form of vitamin D in the blood and circulatory system used to measure levels of vitamin D in the body.

25OHD 1α- hydroxylase enzyme is changed into its active form 1.25 hydroxy-vitamin D (1.25-OHD). Until recently it was thought that 1α- hydroxylase enzyme was only found in the kidneys, but recent studies indicate that 1α- hydroxylase is also found in the brain and other tissues [4, 5]. The latest studies show Vitamin D receptors (VDR) and 1α- hydroxylase enzymes in the central nervous system, in neurons and in glial cells [6]. The strongest immunohistochemical staining for both these receptors and enzymes has appeared in the hypothalamus and within the substantia nigra, suggesting that vitamin D may have autocrine (in other words the activity of vitamin D arises from 1.25OHD synthesized within those cells) and/or paracrine (or 1.25OHD is synthesized in one cell type and acts within adjacent cells) properties in the human brain [6].

Recent studies show that schizophrenia is caused by a neurodevelopmental defect that disrupts early brain formation during specific, but as yet unknown, critical windows of susceptibility [7,8]. Studies of rats with vitamin D deficiencies show histologic and anatomic changes in brain development resulting from vitamin D deficiencies. In particular, studies show narrowing the anterior and posterior cingulate and to the medial and occipital cortical areas [9], enlarged ventricles and shrinking of the hippocampus and the amygdala have also been observed [10]. Histological studies trying to identify the bases of these anatomical changes have examined the dentate gyrus, the hypothalamus, the basal ganglia, the amygdala and the cingulate gyrus and have studied the proliferation of cells, destruction of cells and mitosis in these areas [11].

Vitamin D rich diets or vitamin D supplements have resulted in positive outcomes when added to treatment for some psychiatric illnesses like major depression, Alzheimer's disease, and premenstrual dysphoric syndrome. A correlation between schizophrenia and low levels of vitamin D during the prenatal period and the early childhood have been well demonstrated [12–14]. However, the influence of low vitamin D status on symptom severity in patients with schizophrenia has only recently begun to be investigated. Studies in the literature that compare vitamin D levels and disease severity have obtained varying results. One cross- sectional study that compared severity of symptoms and vitamin D levels in Norwegian and immigrant psychotic patients, showed a correlation between 25OHD levels and severity of negative symptoms like phsycial energy, psychomotor activity, somatic complaints and depressive symptoms results [15]. In a study of patients with schizophrenia experiencing their first psychotic episode, found that vitamin D deficiency facilitated an increase in cognitive deficits and in negative symptoms [16]. Another study identified a moderate negative correlation between vitamin D levels in people diagnosed with schizophrenia and their scores on the Clinical Global Impression–Severity scale and the Positive and Negative Syndrome Scale (PANSS) [17]. On the other hand, a study by Itzhaky et al. comparing the severity of symptoms of people with schizophrenia and major depression with a control group, found no correlation between disease activity, as measured by PANSS scores, and vitamin D levels [18]. Similarly, Crews and colleagues reported that patients with first-episode psychosis have significantly lower concentrations of vitamin D, but they did not find an association between vitamin D concentrations and disease severity [19].

Because the literature still includes very few studies examining the relationship between illness severity and vitamin D levels and those studies show conflicting results, it is necessary to conduct further studies. There are still many questions to be answered. In order to be sure that patients with schizophrenia are properly treated should we be including vitamin D in their treatment? Our study aims to examine the following questions related to this problem: 1) Is there a difference between the levels of vitamin D for individuals diagnosed with schizophrenia and healthy people? 2) Is there a relationship between the positive and negative symptoms of schizophrenia patients and their vitamin D levels? 3) How do lifestyle factors like cigarette smoking and obesity affect vitamin D levels?

## Materials and Methods

### Subject characteristics and procedures

This study included patients 18 to 55 years old admitted to an outpatient clinic between September 2014 and March 2015 who met the criteria for schizophrenia according to DSM-IV TR [20]. They met the Andreasen criteria [21] scoring ≤2 on the SANS and SAPS subscale scores for at least 6 months of remission. Because patients undergoing an acute psychotic episode would not be able to cooperate sufficiently, they were excluded from the study.

Individuals were excluded according to the following factors. A diagnosis of alcohol or substance dependence, organic mental disorders, learning disabilities, or a metabolic disease; those using antiepileptic medications like phenytoin, barbiturates, cholesterol lowering medications or corticosteroids that might affect serum vitamin D concentrations were all excluded. Patients and healthy controls with prior vitamin D supplementation history were also excluded. When these exclusion criteria were applied to 131 admissions, 80 subjects participated in the study. 31 subjects were excluded due to comorbid Axis 1 diagnoses, 4 were excluded due to antiepileptic and cortisol medication usage, 6 subjects due to vitamin D and calcium treatment, 1 subject due to Crohn’s disease, 1 due to intestinal resection, and 8 subjects due to alcohol usage.

Seventy-four age- and sex-matched controls with no diagnosed major psychopathology were recruited from the Physical Medicine and Rehabilitation Department of the Ankara Dıskapı Yıldırım Beyazıt Training and Research Hospital. These healthy subjects had no relevant current or past psychiatric or physical illness as determined by a psychiatric and physical examination and laboratory testing, including a toxicology screening.

This study was approved by Ankara Numune Teaching and Research Hospital ethics committee. Following an explanation of the entire procedure, all participants provided written informed consent for their participation in the study.

All patients were given the Scale for the Assessment of Negative Symptoms (SANS) and the Scale for the Assessment of Positive Symptoms (SAPS). The SANS is a 25 item-scale used to assess five symptom complexes to obtain clinical ratings of negative symptoms in patients with schizophrenia. These include affective blunting, alogia, avolition/apathy, anhedonia/asociality and disturbances of attention. Assessments were conducted on a 6-point scale (0 = not at all to 5 = severe). This instrument was developed by Andreasen [22]. The Turkish version was found to be reliable by Erkoç et al. [23]. The SAPS, a 34- item scale, was designed to assess positive symptoms. This instrument is designed to compliment the SANS. Positive symptoms assessed include hallucinations, delusions, bizarre behavior, and positive formal thought disorders. The SAPS was developed by Andreasen [24]. The Turkish version was found to be reliable by Erkoç et al. [25].

All subjects were given a sociodemographic questionnaire recording age, sex, marital status, weight, height, physical activity, smoking history and a nutritional status assessment. Physical activity was also assessed by questionnaire. Participants were asked to describe how often they performed moderate physical activities like continuous walking, house-work and gardening. They were grouped according to those who reported more than 4 times, 1–3 times and less than one time per week.

Subjects’ body mass index (BMI) was calculated from height and weight measurements. A body mass index of BMI < 18.5 kg/m2 was considered underweight, 18.5–24.9 kg/m2 was considered normal, 25.0–29.9 kg/m2 was considered overweight, and ≥30 kg/m2 was considered obese, in accordance with World Health Organization classifications [26].

### Vitamin D assay methods

Sera from the 80 schizophrenia patient subjects and their 74 matched controls were analyzed for vitamin D levels. All serum samples were biobanked at baseline and stored at −80°C. 25OHD levels depend mainly on sampled individual’s sunlight exposure (cholecalciferol is synthesized in the skin under the impact of ultraviolet light) [27] and exhibit seasonal or monthly variation. Therefore, the time of year, from September to March, in which blood was drawn was the same for the schizophrenia patients and the healthy control group to control for differential effects of sun exposure on vitamin D levels.

The 25OHD values of the participants were measured by electroluminescence with a Roche Cobas E601 analyzer. Vitamin D levels were classified as being sufficient if the 25OHD value was 20–100ng/mL (0.8–4 IU/mL), insufficient if the value was 10–20mg/mL (0.4–0.8 IU/mL), and deficient if the value was <10ng/mL (< 0.4 IU/mL) [3].

### Statistical Methods

The relationships between these schizophrenia patients’ 25OHD levels, SANS and SAPS scores, SANS and SAPS subscale scores and other variables were analyzed using corelational analyses. The comparison of SANS and SAPS total scores among the three groups (deficiency, insufficiency and sufficiency) chosen as the primary analysis, and subsequently, the comparison of each symptom severity proceeded with as the exploratory analyses.

All statistical analyses were performed using SPSS software version 22.0 (SPSS, Chicago, IL). Descriptive analyses are presented here as frequencies, percentages, mean, and standard deviations. Continuous variables were investigated using the Kolmogorov–Smirnov test to determine normal distribution. A chi-square test was used to compare categorical variables in different groups. A Student’s t-test, one-way analysis of variance, or the Kruskal–Wallis test was used to compare continuous variables between the study groups. A Bonferroni and Bonferroni-corrected Mann-Whitney U test was applied in a post hoc analysis for multiple comparisons of the three groups. An adjusted p value with Bonferroni-corrected Mann-Whitney U test of <0.017 was considered statistically significant. A p value of <0.05 was considered statistically significant for the other analyses.

## Results

### Comparison of demographic variables and 25OHD levels between schizophrenia patients and the healthy control groups

The control and schizophrenia diagnosed groups’ sociodemographic characteristics and 25OHD levels are shown in Table 1. There were no significant differences between the patient group and the control group in terms of age, sex or physical activity. An analysis shows statistically significant differences between the groups’ cigarette consumption (p<0.001), BMI (p<0.001) and marital status (p<0.001) **(Table 1).**

**Table 1:** Sociodemographic and clinical characteristics of the patient and control groups.

		Patients (n = 80)	HC (n = 74)	Test statictic	*p*-Value
**Age (years)(X±SD)**		36.59±9.96	35.76±9.01	*t*(152) = 0.541	0.589
**Marital status**					
	**Married**	30 (37.5%)	59 (79.7%)	*χ*^**2**^(4) = 34.826	**<0.001**
	**Single**	39 (48.8%)	12 (16.2%)		
	**Divorced**	11 (13.8%)	3 (4.1%)		
**Sex**					
	**Female**	38(47.5%)	35 (47.3%)	*χ*^**2**^(1) = 0.003	0.956
	**Male**	42(52.5%)	39 (52.7%)		
**Duration of illness (years) (X±SD)**		8.40±7.77	-		-
**Duration of antipsychotic use (month) (X±SD)**		54.08±77.62	-		-
**Smoking (per day) (X±SD)**		15.66±17.45	6.11±8.65	*t*(151) = 4.225	**<0.001**
**Physical activity**					
	**<once per week**	63 (78.8%)	65 (87.8%)	*χ*^**2**^(2) = 3.302	0.192
	**1–3 times per week**	15 (20%)	9 (12.2%)		
	**≥4 times per week**	2 (1.2%)	0 (0%)		
**BMI**					
	**<25 kg/cm**^**2**^	27 (33.8%)	55 (74.3%)	*χ*^**2**^(2) = 26.500	**<0.001**
	**25.0–29.99kg/cm**^**2**^	28 (35%)	13 (17.6%)		
	**≥30 kg/cm**^**2**^	25 (31.2%)	6 (8.1%)		
**25OHD (ng/ml) (X±SD)**		23.46±13.98	23.69±9.61	*t*(152) = -0.122	0.903
**SANS(X±SD)**		36.32±20.07	-		-
**SAPS (X±SD)**		18.79±13.90	-		-

Notes. HC = Healthy controls, BMI = Body mass index,

The mean 25OHD levels of the groups were also similar, 23.46±13.98ng/mL for the patient group and 23.69±9.61ng/mL for the control group. Their average SAPS score was 18.79±13.90 and their average SANS score was 36.32±20.07.

**Fig 1:**
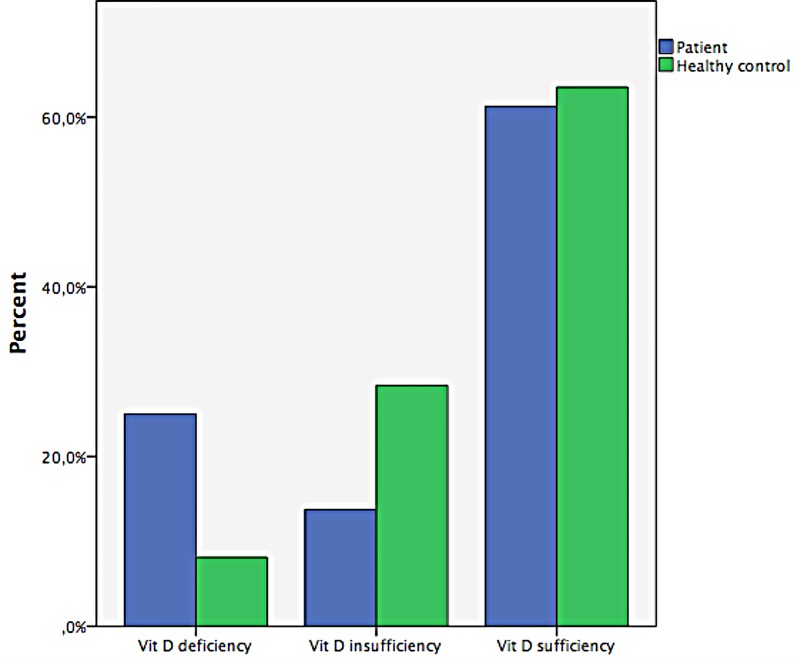
Distribution of Schizophrenia patients according to vitamin D levels.

Among the 80 schizophrenia patients, 25% (n = 20) showed a 25OHD deficiency, 13.75% (n = 11) showed insufficiency and 61.25% showed (n = 49) sufficient levels of 25OHD. Among the 80 healthy controls, 8.1% (n = 6) showed a 25OHD deficiency, 28.4% (n = 21) show insufficiency and 63.5% showed (n = 47) sufficient levels of 25OHD **(Fig 1)**.

We did not identify statistically significant differences in cigarette smoking, physical acitivity, and BMI between the healthy control group and those who were grouped according to deficient, insufficient, and sufficient Vitamin D levels **(Table 2 and Table 3).**

**Table 2:** Comparison of the 25OHD levels of the patient group based on smoking, physical activity, and BMI.

		Deficient <10ng/mL (n = 20)	Insufficient 10-20ng/mL (n = 11)	Sufficient ≥20ng/mL (n = 49)	Test statistic	*p*-Value
**Smoking (per day) (X±SD)**		13.00±18.66	13.00±13.00	17.35±17.91	*χ*^**2**^(2) = 1.665	0.435
**Physical activity**						
	**<once per week**	17 (85%)	7 (63.7%)	39 (79.6%)	*χ*^**2**^(4) = 3.812	0.432
	**1–3 times per week**	3 (15%)	4 (36.3%)	8 (16.3%)		
	**≥4 times per week**	0 (0%)	0 (0%)	2 (4.1%)		
**BMI**						
	**<25 kg/cm**^**2**^	7 (35%)	2 (18.2%)	18 (36.7%)	*χ*^**2**^(4) = 1.711	0.789
	**25.0–29.9 kg/cm**^**2**^	6 (30%)	5 (45.5%)	17 (34.7%)		
	**≥30 kg/cm**^**2**^	7 (35%)	4 (36.4%)	14 (28.6%)		

Notes. BMI = Body mass index

**Table 3:** Comparison of the 25OHD levels of the healthy control group based on smoking, physical activity, and BMI.

		Deficient <10ng/mL (n = 6)	Insufficient 10–20ng/mL (n = 21)	Sufficient ≥20ng/mL (n = 47)	Test statictic	*p*-Value
**Smoking (per day) (X±SD)**		6.67±12.11	6.20±9.66	6.00±7.91	*χ*^**2**^(2) = 0.817	0.911
**Physical activity**						
	**<once per week**	5 (%83.3)	19 (%90.5)	41(%87.2)	*χ*^**2**^(2) = 0.267	0.875
	**1–3 times per week**	1 (%16.7)	2 (%9.5)	6 (%12.8)		
	**≥4 times per week**	0 (0%)	0 (0%)	0 (0%)		
**BMI**						
	**<25 kg/cm**^**2**^	4 (%66.6)	19 (%90.4)	32 (%68.1)	*χ*^**2**^(4) = 4.746	0.314
	**25.0–29.9 kg/cm**^**2**^	1 (%16.7)	1 (%4.8)	11 (%23.4)		
	**≥30 kg/cm**^**2**^	1 (%16.7)	1 (%4.8)	4 (%8.5)		

Notes. BMI = Body mass index

### Vitamin D status and symptom severity in patients with schizophrenia

A statistically significant differences was also found when the schizophrenia patients were grouped according to their 25OHD levels and their SANS total, affective flattening or blunting, their SAPS total, bizarre behavior and positive formal thought disorders scores: in order p = 0.019, p = 0.004, p = 0.015, p = 0.009 and p = 0.019).

Triple analyses were analyzed post hoc and statistically significant differences were found between scores for deficient and sufficient SANS totals (p = 0.008), affective flattening or blunting (p = 0.037), SAPS totals (p = 0.017) and positive formal thought disorders (p = 0.007). Differences were also identified in bizarre behavior scores between deficient and sufficient (p = 0.013), and deficient and insufficient groups (p = 0.006). (Table 4).

**Table 4:** Relationship between 25OHD levels and SANS and SAPS scores.

	Deficient <10ng/mL (n = 20)	Insufficient 10-20ng/mL (n = 11)	Sufficient ≥20ng/mL (n = 49)	Test statictic	*p*-Value
**SANS**	46.55±22.31	36.55±15.20	32.10±18.86	*χ*^**2**^(2) = 7.967^*a^	0.019
**AF or Blunting**	13.95±8.08	9.63±8.47	8.97±6.67	F(2) = 3.349^*b^	0.040
**Alogia**	6.60±4.19	6.27±4.54	5.08±3.85	F(2) = 1.169	0.316
**Avolition-Apathy**	6.80±4.08	5.09±3.72	4.61±3.37	*χ*^**2**^(2) = 2.811	0.079
**Anhedonia- Asociality**	11.00±6.93	8.72±6.08	7.87±5.50	*χ*^**2**^(2) = 3.899	0.245
**Attention**	8.50±5.32	7.81±3.97	6.14±3.40	F(2) = 4.420^*c^	0.142
**SAPS**	26.45±18.63	15.45±10.02	16.41±11.27	*χ*^**2**^(2) = 0.839	0.015
**Hallucinations**	4.30±6.25	2.18±3.57	2.63±3.68	*χ*^**2**^(2) = 0.146	0.658
**Delusions**	7.05±8.29	6.27±6.16	5.02±3.81	*χ*^**2**^(2) = 9.382^*d^	0.930
**Bizarre behavior**	6.25±3.99	2.54±1.75	3.83±2.81	*χ*^**2**^(2) = 7.941^*e^	0.009
**PFTD**	8.80±5.56	7.09±10.09	5.06±3.40	*χ*^**2**^(2) = 2.811	0.019

Notes. AF = Affective flattening, PFTD = positive formal thought disorder

*a:significant between deficient and sufficient group (p = 0.008)

*b:significant between deficient and sufficient group(p = 0.037)

*c:significant between deficient and sufficient group (p = 0.017)

*d:significant between deficient and sufficient group (p = 0.013), significant between deficient and insufficient group (p = 0.006)

*e:significant between deficient and sufficient group (p = 0.007)

### Correlation analyses

We found a statistically significant negative correlation between 25OHD levels and SANS total points (r = -0.232, p = 0.038); a statistically significant negative correlation for attention points (r = -0.227, p = 0.044); and a significant negative correlation with positive formal thoughts (r = -0.257, p = 0.021). We did not find a correlation between 25OHD, cigarette smoking and BMI.

## Discussion

In our study, when patients with and without vitamin D deficiency were compared, we found a statistically significant difference between their SANS and SAPS total scores and some subscale scores, such as affective flattening, bizarre behavior, and positive formal thought. To our knowledge, this study is the first to find a relationship between positive symptoms and vitamin D deficiency. A result that drew our attention was that greater levels of vitamin D deficiency corresponded with higher scores on the SANS and SAPS scales. Of the schizophrenia patients who participated in our study, 20% showed deficient levels of vitamin D; 13.75% were found to have insufficient levels; and 61.25% were found to have sufficient levels. The healthy controls in our study showed 8.1% deficient, 28.4% insufficient and 63.5% sufficient levels of Vitamin D. Although the deficient percentage among the schizophrenia patients was higher, the levels found for vitamin D in our schizophrenia patients and healthy controls was not statistically significant.

The literature shows different results in for vitamin D levels in schizophrenia patients. One study, like ours, found similar levels of vitamin D between healthy controls and schizophrenia patients 16. Other studies have found lower levels of vitamin D in schizophrenia patients vs controls [18, 19, 28]. A study compared a group of schizophrenia patients in remission with a group of healthy controls and a group experiencing a first acute exacerbation of the illness. This study found that levels of vitamin D were lower in the group of schizophrenia patients experiencing acute exacerbation episodes. This study also found patients in remission and healthy controls had similar vitamin D levels [17]. Berg et al. conducted a cross-sectional study comparing healthy Norwegians, and Norwegians and immigrants experiencing psychosis. This study identified lower levels of vitamin D in the schizophrenia patient group in comparison to controls but also showed that levels were lowest in the immigrant psychotic patient group [15].

The differing results obtained in these studies could be the result of factors like darker complexions and geographic differences in where the studies were conducted. Other factors may also make a difference for instance differences in phases of the disorder like acute or chronic stages. Also differences were observed between patients experiencing acute exacerbation and those in remission. Greater levels of negative and positive symptoms may increase nutritional problems, reduce the amount of exposure to sunlight, and encourage more sedentary lifestyle habits. This may also account for lower levels of vitamin D in patients undergoing acute episodes of schizophrenia when compared with more similar levels in those in remission and in controls. It is also possible that the different scores could be a result of taking blood samples during different seasons of the year. Studies done by Graham et al. and Yüksel et al. [16,17] like ours, took care to gather blood samples during the same season. This could be a reason for the similar results obtained by our study and these others.

Animal studies have shown that vitamin D takes a very active role in healthy brain functioning [6]. In patients with schizophrenia, vitamin D most affects brain function in the following regions: the hippocampus, the thalamus, the hypothalamus, the amygdala, the prefrontal cortex and the temporal lobe. Many studies show evidence of a correlation between the development of schizophrenia and low prenatal and early life vitamin D status [12,14]. It has been hypothesized that low vitamin D levels may impair neurogenesis by decreasing protein kinase B levels, which are important for neuronal growth, differentiation, and migration in schizophrenia. It has been reported that apparently, vitamin D prevents neuronal free calcium uptake and, consequently, cellular hyperpolarization and its toxic actions in fetal hippocampal neurons [29]. This finding probably relates to the fact that vitamin D decreases the number of L-type voltage-gated calcium channels thus reducing calcium influx [30]. L-type voltage-gated calcium channels have been shown to play a role in behaviors mediated by the mesolimbic pathway and amygdala [31].

Vitamin D deficiency is thought to cause memory and attention deficits by reducing GABAergic and glutamatergic neurotransmissions, which are important for attention and working memory in the dorsolateral prefrontal cortex. Therefore, vitamin D deficiency could theoretically also lead to reduced dorsolateral dopaminergic trasmission, which may lead to attention and memory deficits [32]. While we may not yet understand its effects on the glutamate system, vitamin D’s close relationship to the dopaminergic system has been shown [33, 34]. Laurelle et al. showed reduced transmission by N-methyl-D-aspartate receptors (NMDA) in the prefrontal cortex resulting in problems in cognitive functions and reductions in mesocorticolimbic dopamine transmissions [35]. Reduced NMDA transmission may cause both a cortical dopamine deficit and excess associative striatum dopamine [35]. Given that the glutaminergic system is closely associated with the dopaminergic system, there is likely to be a close relationship between vitamin D and glutaminergic transmission [36]. Vitamin D deficiency also leads to hyperlocomotion in response to NMDA-antagonists [33, 37–39] and is reverted by haloperidol [37].

In our study, when patients with and without vitamin D deficiency were compared, there was a statistically significant difference between their SANS and SAPS total scores and some subscale scores, such as affective flattening, bizarre behavior, and positive formal thought. In addition, there was a statistically significant negative correlation between 25OHD levels and SANS total points, attention points and positive formal thought points. As a result of vitamin D deficiency, positive symptoms such as disorganized speech and disorganized behavior could also be seen in addition to symptoms of attention and memory deficit. Our findings support previous research results for the Vitamin D deficiency model.

Associations between Vitamin D deficiency and obesity, smoking, and lack of physical activity have been shown in previous studies [40–43] and we analyzed those variables in the current study as well. Although there were significant differences between healthy controls and our patient group in terms of obesity and smoking, there was no statistically significant difference in their vitamin D values. Our study did not look carefully at nutrition and patients who took supplemental vitamin D. We know that, other than supplements, sunlight exposure is the primary source of vitamin D for the majority of people [44]. In our study, the similarity of vitamin D levels, despite large statistical differences between our schizophrenia patient group and controls in relation to smoking and obesity, supports the idea that sunlight is a very important factor. When patients with schizophrenia were grouped according to their levels of vitamin D deficiency, there were no statisticallysignificant differences in terms of obesity and smoking between the groups. This finding strengthens the finding of a relationship between vitamin D deficiency and symptom severity.

We know that cigarette smoking and BMI are the factors that affecting vitamin D levels [42]. We have done partial corelation analysis between BMI and cigarettte smoking with the smoking states using as controlling factor. This analyses revealed that there was a a statistically significant negative correlation between 25OHD levels and SANS total points (r = -0.258, p = 0.023); a statistically significant negative correlation for SAPS total points (r = - 0.250, p = 0.028); a statistically significant negative correlation for attention points (r = - 0.257, p = 0.024); a statistically significant negative correlation for avolution points (r = -0.260, p = 0.023); a statistically significant negative correlation for bizarre behavior points (r = -0.248, p = 0.029) and a significant negative correlation with positive formal thoughts (r = -0.276, p = 0.015). We did not find a correlation between 25OHD, cigarette smoking and BMI.

The limitations of our study include the fact that we did not examine the relationship between negative symptoms and application of cognitive tests, and consider different phototypes. The classification of skin phototypes (type I to VI) has been based on the ability of individuals with different constitutive pigmentation to burn or tan in response to sun exposure. Skin phototype I represents individuals with very fair skin who always burn and never tan when exposed to the sun. Skin phototype II individuals tan minimally with difficulty and burn easily, skin phototype III individuals tan moderately and uniformly and burn moderately while type IV individuals burn minimally and tan moderately and easily. Finally, skin phototype V represent individuals with very dark skin who rarely burn and tan profusely, while type VI individuals never burn and tan profusely upon sun exposure [45]. Though our study did not include racially dark skinned individuals (phototype 6), there may nevertheless be differences in the vitamin D levels of individuals with phototypes 1 through 5, producing erroneous results. By taking study participants' blood samples between September and March we attempted to reduce seasonal differences in vitamin D levels. Taking samples during one time of year or even during one month reduces this kind of difference. Our study worked with subjects who met the Andreasen criteria for remission of schizophrenia. According to theory it is to be expected that patients in remission will have SANS and SAPS scores lower than those undergoing an acute episode. It is possible that those with more severe symptoms would have a differing relationship between the severity of their symptoms and their levels of vitamin D. We can consider these potentially missing factors in our study. And also the limitation of including only remitted patients narrowed the range of the severity of the subjects, which may have in turn influenced the statistics. We did not assessed the adverse effects in the patient population. This may be another limitation of our study. It is also possible that studying both sexes together may be a limitation. To study each sex separately would remove sex as a confounding factor. Further studies that include patients in both the acute and remission phases, that consider only one sex, involve greater numbers of subjects, and that study all of the variables that affect vitamin D levels are needed.

In conclusion, we suggest that it is necessary to evaluate the levels of vitamin D in schizophrenia patients. Our results, show that lower vitamin D levels were related to positive and negative symptom severity and as the vitamin deficiency became greater, patients’ symptom scores increased, suggesting that it is advisable to monitor the vitamin D levels of patients being treated for schizophrenia. An impact on response to treatment from the addition of vitamin D3 during a first episode or recurrent episodes is an important finding. As of now, changes in disease severity and course resulting from the addition of vitamin D to treatment are unknown, as is the required dose of vitamin supplementation for these cases. Based upon these findings, future trials are needed that focus on dosing and toleration of vitamin D supplementation with patients diagnosed with schizophrenia.

## References

[1] McGrath J. Hypothesis: is low prenatal vitamin D a risk-modifying factor for schizophrenia? Schizophr Res. 1999; 40 (3):173–177. 10638855 10.1016/s0920-9964(99)00052-3

[2] Wrzosek M, Lukaszkiewicz J, Wrzosek M, Jakubczyk A, Matsumoto H, Piatkiewicz P, et al Vitamin D and the central nervous system. Pharmacol Rep. 2013; 65 (2): 271–278. 23744412 10.1016/s1734-1140(13)71003-x

[3] Holick M. Vitamin D deficiency. N Engl J Med. 2007; 357(3): 266–281. 10.1056/NEJMra070553 17634462

[4] Garcion E, Wion-Barbot N, Montero-Menei CN, Berger F, Wion D. New clues about vitamin D functions in the nervous system. Trnds endocrinol Metab. 2002; 13(3):100–105. 10.1016/s1043-2760(01)00547-1 11893522

[5] Nimitphong H, Holick MF. Vitamin D, neurocognitive functioning and immunocompetence. Curr Opin Clin Nutr Metab Care. 2011;14(1): 7–14. 10.1097/MCO.0b013e3283414c38 21102318

[6] Kalueff A, Tuohimaa P. Neurosteroid hormone vitamin D and its utility in clinical nutrition. Curr Opin Clin Nutr Metab Care. 2007;10(1): 12–19. 10.1097/MCO.0b013e328010ca18 17143049

[7] Meli G, Öttl B, Paladini A, Cataldi L. Prenatal and perinatal risk factors of schizophrenia. J Matern Fetal Neonatal Med. 2012; 25(12): 2559–2563. 10.3109/14767058.2012.699118 22646662

[8] Meyer U, Feldon J. Epidemiology-driven neurodevelopmental animal models of schizophrenia. Prog Neurobiol. 2010; 90 (3): 285–326. 10.1016/j.pneurobio.2009.10.018 19857543

[9] Narr KL, Toga AW, Szeszko P, Thompson PM, Woods RP, Robinson D, et al Cortical thinning in cingulate and occipital cortices in first episode schizophrenia. Biol Psychiatry. 2005; 58(1): 32–40. 10.1016/j.biopsych.2005.03.043 15992520

[10] Lawrie S, Abukmeil S. Brain abnormality in schizophrenia. A systematic and quantitative review of volumetric magnetic resonance imaging studies. Br J Psychiatry. 1998; 172: 110–120. 9519062 10.1192/bjp.172.2.110

[11] Eyles D, Brown J, Mackay-Sim A, McGrath J, Feron F. Vitamin D3 and braindevelopment. Neuroscience. 2003; 118 (3): 641–653. 12710973 10.1016/s0306-4522(03)00040-x

[12] McGrath J, Saari K, Hakko H, Jokelainen J, Jones P, Järvelin MR, et al Vitamin D supplementation during the first year of life and risk of schizophrenia: a Finnish birth cohort study. Schizophr Res. 2004; 67(2): 237–245. 14984883 10.1016/j.schres.2003.08.005

[13] McGrath JJ, Burne TH, Feron F, Mackay-Sim A, Eyles DW. Developmental Vitamin D deficiency and risk of schizophrenia: a ten-year update. Schizophr Bull. 2011;36: 1073–1078. 10.1093/schbul/sbq101 PMC2963051 20833696

[14] Torrey EF, Torrey BB, Peterson MR. Seasonality of schizophrenic births in the United States. Arch Gen Psychiatry. 1977; 34(9): 1065–1070. 901136 10.1001/archpsyc.1977.01770210079007

[15] Berg AO, Melle I, Torjesen PA, Lien L, Hauff E, Andreassen OA. A cross-sectional study of vitamin D deficiency among immigrants and Norwegians with psychosis compared to the general population. J Clin Psychiatry. 2010; 71(12):1598–1604. 10.4088/JCP.09m05299yel 20441728

[16] Graham KA, Keefe RS, Lieberman JA, Calikoglu AS, Lansing KM, Perkins DO. Relationship of low vitamin D status with positive, negative and cognitive symptom domains in people with first- episode schizophrenia. Early Interv Psychiatry. 2014 10.111/eip.12122 “In press” 24612563

[17] Yuksel RN, Altunsoy N, Tikir B, Külük MC, Unal K, Goka S, et al Correlation between total vitamin D levels and psychotic psychopathology in patients with schizophrenia: therapeutic implications for add-on vitamin D augmentation. Ther Adv Psychopharmacol. 2 2014; 4 (6): 268–275. 10.1177/2045125314553612 25489478 PMC4257987

[18] Itzhaky D, Amital D, Gorden K, Bogomolni A, Arnson Y, Amital H. Low serum vitamin D concentrations in patients with schizophrenia. Isr Med Assoc J. 2012; 14 (2): 88–92. 22693787

[19] Crews M, Lally J, Gardner- Sood P, Howes O, Bonaccorso S, Smith S, et al Vitamin D deficiency in first episode psychosis: a case–control study. Schizophr Res. 2013;150(2–3): 533–537. 10.1016/j.schres.2013.08.036 24060571

[20] American Psychiatric Association. Diagnostic and Statistical Manual of Mental Disorders, 4th Edition, Text Revision (DSM-IV-TR); 2000.

[21] Andreasen NC, Carpenter WT Jr, Kane JM, Lasser RA, Marder SR, Weinberger DR. Remission in schizophrenia: proposed criteria and rationale for consensus. Am J Psychiatry. 2014;162: 441–449. 10.1176/appi.ajp.162.3.441 15741458

[22] Andreasen NC. Scale for the assesment of negative symptoms: SANS Lowa: Dept. of Psychiatry, College of Medicine, the University of Lowa 1984.

[23] Erkoç S, Arkonaç O, Ataklı C, Ozmen E. The validity and reliability of the scale for the assessment of negative symptoms (in Turkish). Dusunen Adam. 1991; 4: 16–19.

[24] Andreasen NC. Scale for the assesment of positive symptoms: SAPS Iowa: Dept. of Psychiatry, College of Medicine, the University of Iowa 1984.

[25] Erkoç S, Arkonaç O, Ataklı C, Ozmen E. The validity and reliability of the scale for the assessment of positive symptoms (in Turkish). Dusunen Adam 1991; 4: 20–24.

[26] WHO Expert Consultation. Appropriate body-mass index for Asian populations and its implications for policy and intervention strategies. Lancet. 2004; 363 (9403): 157–163. 10.1016/S0140-6736(03)15268-3 14726171

[27] Devgun MS, Paterson CR, Johnson BE, Cohen C. Vitamin D nutrition in relation to season and occupation. Am J Clin Nutr. 1981;34(8): 1501–1504. 7270473 10.1093/ajcn/34.8.1501

[28] Jamilian H, Bagherzadeh K, Nazeri Z, Hassanijirdehi M. Vitamin D, parathyroid hormone, serum calcium and phosphorus in patients with schizophrenia and major depression. Int J Psychiatry Clin Pract. 2013;17(1):30–34. 10.3109/13651501.2012.667111 22536888

[29] Eyles D, Burne T, McGrath J. Vitamin D in fetal brain development. Semin Cell Dev Biol. 2011; 22(6): 629–636. 10.1016/j.semcdb.2011.05.004 21664981

[30] Brewer LD, Thibault V, Chen KC, Langub MC, Landfield PW, Porter NM. Vitamin D hormone confers neuroprotection in parallel with downregulation of L-type calcium channel expression in hippocampal neurons. J Neurosci. 2001;21(1): 98–108. 11150325 10.1523/JNEUROSCI.21-01-00098.2001 PMC6762438

[31] Bhat S, Dao DT, Terrillion CE, Arad M, Smith RJ, Soldatov NM, et al CACNA1C (Cav 1.2) in the pathophysiology of psychiatric disease. Prog Neurobiol. 2012; 99(1): 1–14. 10.1016/j.pneurobio.2012.06.001 22705413 PMC3459072

[32] Lewis DA, Gonzalez-Burgos G. Pathophysiologically based treatment interventions in schizophrenia. Nat Med. 2006; 12(9): 1016–1022. 10.1038/nm1478 16960576

[33] Kesby JP, Cui X, O'Loan J, McGrath JJ, Burne TH, Eyles DW. Developmental vitamin D deficiency alters dopamine-mediated behaviors and dopamine transporter function in adult female rats. Psychopharmacology (Berl). 2010; 208(1):159–168. 19921153 10.1007/s00213-009-1717-y

[34] Kahn RS, Harvey PD, Davidson M, Keefe RS, Apter S, Neale JM, et al Neuropsychological correlates of central monoamine function in chronic schizophrenia: relationship between CSF metabolites and cognitive function. Schizophr Res. 1994; 11(3): 217–224. 7514887 10.1016/0920-9964(94)90015-9

[35] Laruelle M. Schizophrenia: from dopaminergic to glutamatergic interventions. Curr Opin Pharmacol. 2014; 14: 97–102. 10.1016/j.coph.2014.01.001 24524997

[36] Amaral AD, Calhau C, Coelho R. Schizophrenia: implications of vitamin D deficit on brain development. IJCNMH. 2014; 1(16): 1–14.

[37] Kesby JP, Burne THJ, McGrath JJ, Eyles DW. Developmental vitamin D deficiency alters MK 801-induced hyperlocomotion in the adult rat: An animal model of schizophrenia. Biol Psychiatry. 2006; 60(6):591–596. 10.1016/j.biopsych.2006.02.033 16697353

[38] Kesby JP, O'Loan JC, Alexander S, Deng C, Huang XF, McGrath JJ, et al Developmental 5 vitamin D deficiency alters MK-801-induced behaviours in adult offspring. 6 Psychopharmacology (Berl). 2012; 220(3): 455–463. 21947313 10.1007/s00213-011-2492-0

[39] O'Loan J, Eyles DW, Kesby J, Ko P, McGrath JJ, Burne THJ. Vitamin D deficiency during various stages of pregnancy in the rat; its impact on development and behaviour in adult offspring. Psychoneuroendocrinology. 2007; 32(3): 227–234. 10.1016/j.psyneuen.2006.12.006 17276604

[40] Buffington C, Walker B, Cowan GS Jr, Scruggs D. Vitamin D deficiency in the morbidly obese. Obes Surg. 1993; 3(4): 421–424. 10.1381/096089293765559142 10757956

[41] Gerdhem P, Ringsberg KAM, Obrant KJ, Akesson K. Association between 25-hydroxy vitamin D levels, physical activity, muscle strength and fractures in the prospective population-based OPRA Study of Elderly Women. Osteoporos Int. 2005;16(11): 1425–1431. 10.1007/s00198-005-1860-1 15744449

[42] Kassi EN, Stavropoulos S, Kokkoris P, Galanos A, Moutsatsou P, Dimas C, et al Smoking is a significant determinant of low serum vitamin D in young and middle-aged healthy males. Hormones (Athens) 2014 10.14310/horm.2002.1521 “In press” 25402376

[43] Wortsman J, Matsuoka LY, Chen TC, Lu Z, Holick MF. Decreased bioavailability of vitamin D in obesity. Am J Clin Nutr. 2000; 72(3): 690–693. 10966885 10.1093/ajcn/72.3.690

[44] Calvo MS, Whiting SJ, Barton CN. Vitamin D intake: av global perspective of current status. J Nutr. 2005; 135(2): 310–316. 15671233 10.1093/jn/135.2.310

[45] Sachdeva S. Fitzpatrick skin typing: applications in dermatology. Indian J Dermatol Venereol Leprol. 2009; 75(1): 93 19172048 10.4103/0378-6323.45238

